# Cellular and Mucosal Immune Responses Following Vaccination with Inactivated Mutant of *Escherichia coli* O157:H7

**DOI:** 10.1038/s41598-019-42861-z

**Published:** 2019-04-22

**Authors:** Robert G. Schaut, Paola M. Boggiatto, Crystal L. Loving, Vijay K. Sharma

**Affiliations:** 1 USDA-ARS, National Animal Disease Center, Ames, IA USA; 2 Food Safety and Enteric Pathogens Research Unit, Ames, IA USA; 3 Oak Ridge Institute for Science and Education (ORISE), ARS Research Participation Program, Oak Ridge, TN USA; 4 Infectious Bacterial Diseases Research Unit, Ames, IA USA

**Keywords:** Cellular immunity, Humoral immunity, Cell vaccines

## Abstract

Shiga toxin-producing *Escherichia coli* O157:H7 (O157) can cause mild to severe gastrointestinal disease in humans. Cattle are the primary reservoir for O157, which colonizes the intestinal tract without inducing any overt clinical symptoms. Parenteral vaccination can reduce O157 shedding in cattle after challenge and limit zoonotic transmission to humans, although the impact of vaccination and vaccine formulation on cellular and mucosal immune responses are undetermined. To better characterize the cattle immune response to O157 vaccination, cattle were vaccinated with either water-in-oil-adjuvanted, formalin-inactivated *hha* deletion mutant of Shiga toxin 2 negative (stx2^−^) O157 (Adj-Vac); non-adjuvanted (NoAdj-Vac); or non-vaccinated (NoAdj-NoVac) and peripheral T cell and mucosal antibody responses assessed. Cattle in Adj-Vac group had a higher percentage of O157-specific IFNγ producing CD4^+^ and γδ^+^ T cells in recall assays compared to the NoAdj-Vac group. Furthermore, O157-specific IgA levels detected in feces of the Adj-Vac group were significantly lower in NoAdj-Vac group. Extracts prepared only from Adj-Vac group feces blocked O157 adherence to epithelial cells. Taken together, these data suggest parenteral administration of adjuvanted, inactivated whole-cell vaccines for O157 can induce O157-specific cellular and mucosal immune responses that may be an important consideration for a successful vaccination scheme.

## Introduction

Shiga toxin-producing *Escherichia coli* O157:H7 (O157) is a causative agent of hemorrhagic diarrhea, which can progress to fatal hemolytic uremic syndrome (HUS), a condition characterized by hemolytic anemia, acute renal failure, and thrombocytopenia^1^. Many incidents of O157 infections are foodborne; however, environmental exposures through swimming or animal interactions are also important means of transmission^2–8^. Although O157 infections are a reportable disease, cases are likely under-reported due to mild symptoms or asymptomatic individuals^9^. Despite efforts of safe food handling and animal management, the Centers for Disease Control and Prevention (CDC) estimates that 265,000 Shiga toxigenic *E*. *coli* (STEC) infections (36% O157:H7 serogroup-related) occur each year in the United States^10^. Furthermore, conservative estimates suggest that globally 2,801,000 acute illnesses associated with STEC occur annually^11^.

A plausible method for reducing the transmission of O157 to humans is to limit colonization and fecal shedding from the carrier animal species, particularly cattle, which are considered a major reservoir of O157. Cattle can be naturally colonized with O157, do not exhibit overt clinical signs of disease, and can shed variable levels of O157 in their feces. Since fecal contamination of food and water are the main sources of infections for humans^12^, intervention strategies to reduce O157 shedding from cattle have been explored and vaccine approaches include subunit vaccination targeting flagellin^13^, type 3 secretion system (T3SS)^13–16^, iron acquisition proteins and porins^17^, or as in our previous studies via inactivated, modified whole-cell vaccines^18–22^.

Previously, we demonstrated that deletion of *hha* (Δ*hha*) deregulates *ler* leading to the overexpression of the locus of enterocyte effacement (LEE) encoded T3SS and virulence proteins (T3SP)^23^. This modification would allow for the LEE controlled proteins, which are important in O157 colonization, to become a dominant set of proteins to serve as an immunogenic vaccine-directed targets. Parenteral administration of the heat-killed Δ*hha* construct as a vaccine reduced the duration of O157 fecal shedding by half after experimental challenge compared to non-vaccinated controls (6 vs 12 days, respectively)^18^. Furthermore, the oil-in-water adjuvanted-Δ*hha* vaccination scheme was successful in the reduction of fecal shedding by approximately 2 orders of magnitude^19^. Serum IgG specific to the Δ*hha* vaccine strain was detected, though the specific immune mechanisms contributing to a reduction in O157 fecal shedding post-vaccination are unknown^19^. It is likely that vaccine-directed mucosal IgA^24^ or cellular O157-directed IFNγ^25^ may play an important role in the beneficial immunological response associated with reduced fecal O157 shedding.

IgA and antigen-specific immune cells of the gut-associated lymphoid tissue (GALT) are important in driving a host response toward invasive bacterial species^26,27^. Mucosal secretory IgA is a key component of intestinal defenses, acting as a barrier by blocking bacterial adherence and translocation across the epithelium^28–31^. Likewise, cell-mediated responses at the mucosa may play a role in controlling bacterial attachment. CD4^+^ T cells within the gut mucosa and associated lymph nodes of cattle respond to pathogenic bacterial strains of *E*. *coli*^32^. Human peripheral CD4^+^ T cell responses after vaccination against pathogenic *E*. *coli* demonstrate a robust IFNγ-directed response wherein the authors surmise that the peripheral cells may represent the T cells present within the mucosa^33^. Likewise, γδ T cells may play a role in supporting a productive immune response to O157 as (*E*)-4-hydroxy-3-methyl-but-2-enyl pyrophosphate (HMB-PP), a metabolite of O157, activates γδ T cells to secrete IFNγ and IL-17 leading to increased activation of macrophages and bacterial killing^34^. The IFNγ driven proinflammatory response alongside antigen uptake and processing of *E*. *coli* by peripheral γδ T cells^35^ may be an important mechanism of productive immunological response to O157. It is likely that both humoral and cellular driven responses against pathogenic *E*. *coli* play important roles in limiting colonization.

The goal of this study was to characterize both cellular and mucosal responses directed against O157 after parenteral vaccination with Δ*hha* mutant, using samples collected during a prior study^19^. Given the efficacy of the vaccine, the characterizations can provide insight on desired immune responses after vaccination, guiding future vaccine formulations and antigen targets.

## Results

### Reduced circulating IgM^+^ B cell and γδ CD8^+^ T cells at 6 weeks post-vaccination

To determine any effect of vaccination on circulating immune cell populations, flow cytometry-based phenotyping of peripheral blood mononuclear cells (PBMC) was performed and analyzed with a specific cell gating strategy (Supplemental Fig. 1A). Overall, most immune cell populations did not vary significantly over the course of vaccination (Supplemental Fig. 1B). However, at 6 weeks post-vaccination we did observe a significant difference (*p* < 0.01) in the total number of B cells (determined by surface IgM staining) circulating in the Adj-Vac group (mean count 224 cells/µL) compared to NoAdj-Vac and NoAdj-NoVac control groups (mean count of 394 and 404 cells/µL respectively). The number of circulating CD8^+^ γδ^+^ cells was also significantly reduced (*p* < 0.01) at week 6 in the Adj-Vac group (mean 180 cells/µL) compared to NoAdj-Vac (mean 636 cells/µL) and NoAdj-NoVac controls (mean count 589 cells/µL) (Supplemental Fig. 1B, lower right and lower left panels; solid black line/black circles).

### T cells from Adj-Vac group responded to O157 antigen by proliferating and producing interferon-γ

**Figure 1:**
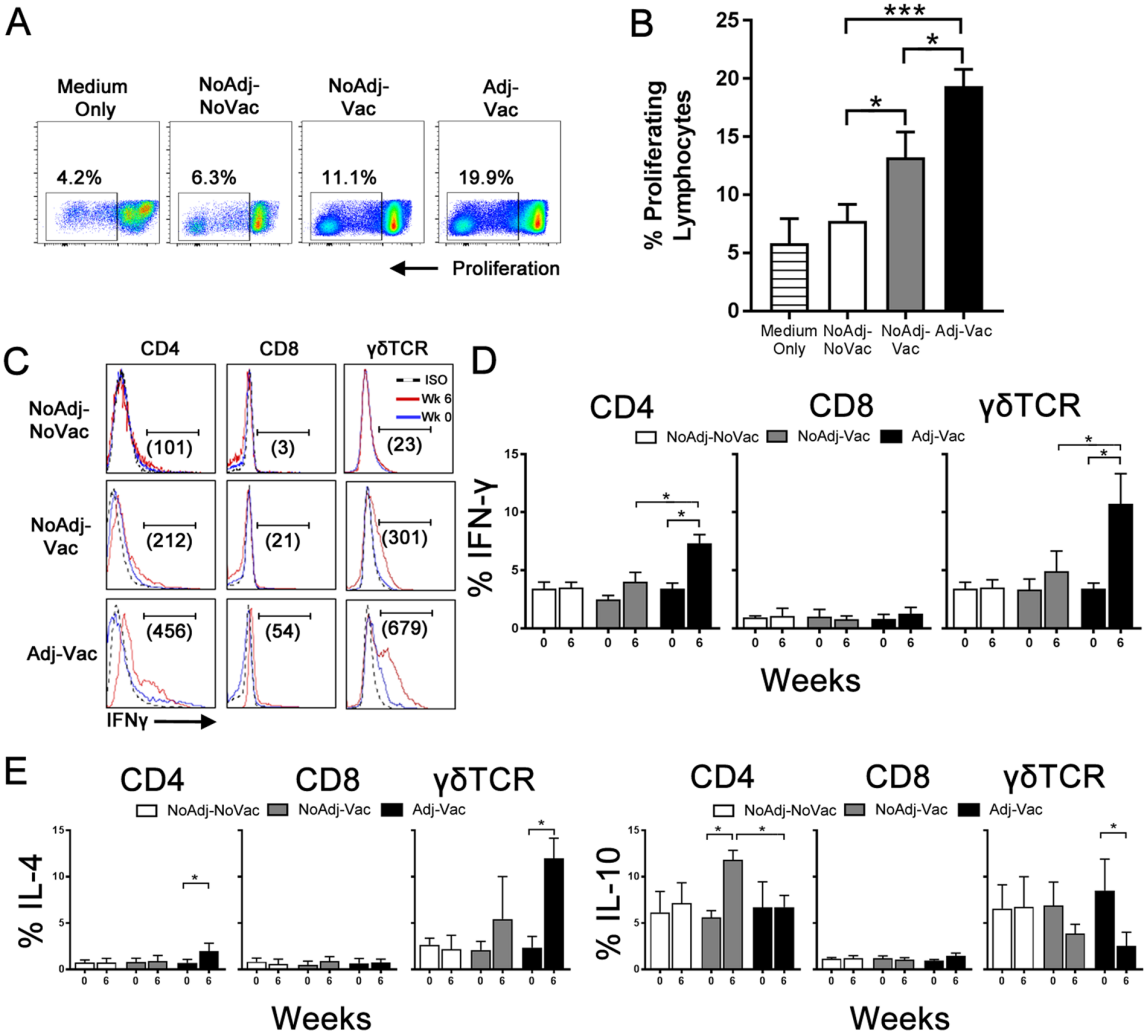
Adjuvant-Vaccination primes an IFN-γ-biased cellular immune response to O157 antigen. (**A**) Representative plots of total lymphocyte proliferation in response to either medium only or O157 antigen stimulation. (**B**) Graphical representation of percentages of proliferating lymphocytes to O157 antigen. (**C**) Intracellular interferon-γ production demonstrated by representative plots for different PBMC types isolated from animals that were given the mock-vaccine (NoAdj-NoVac, top panels), non-adjuvanted vaccine (NoAdj-Vac, middle panels), or adjuvanted vaccine (Adj-Vac, bottom panel). Cells were gated on CD4^+^ (left panels), CD8^+^ (middle panels), or γδ^+^ cells (right panels). The plots represent cell types (as indicated) stimulated for 3 days with 5 μg of O157 antigen. Numbers in parenthesis are differences in IFN-γ ΔMFI (isotype MFI corrected) between weeks 0 and 6. Dashed lines indicate isotype control, solid red indicate week 0 samples, solid blue represents week 6 samples. (**D**) Graphical representation of the data shown in (**C**). Open bars are representive of cells isolated from mock treated animals (NoAdj-NoVac), grey bars are cells from non-adjuvanted, vaccinated animals (NoAdj-Vac) and black bars are cells from adjuvanted, vaccinated (Adj-Vac) animals. Bars represent percentage of intracellular positive IFN-γ Bars are +/−SD. *p < 0.01. Dashed bar represent cells mock-stimulated with medium only, open bars are representive of cells isolated from mock treated animals (NoAdj-NoVac), grey bars are cells from non-adjuvanted, vaccinated animals (NoAdj-Vac) and black bars are cells from adjuvanted, vaccinated (Adj-Vac) animals. (**E**) Differential cytokine responses to O157 antigen stimulation drive lower percentage of IL-10 and greater IL-4 in cells isolated from adjuvanted-vaccinated animals. CD4^+^, CD8^+^ or γδ^+^ intracellular-cytokine responses to O157 antigen stimulation. Cells were stimulated for 3 days with 5 μg of O157 antigen. Open bars are representive of cells isolated from mock treated animals (NoAdj-NoVac), grey bars are cells from non-adjuvanted, vaccinated animals (NoAdj-Vac) and black bars are cells from adjuvanted, vaccinated (Adj-Vac) animals. Bars are +/−SD. *p < 0.01. Bars are +/−SD. **p < 0.001, ***p < 0.0001.

To assess if vaccination schemes elicited O157-specific cell-mediated immune responses, we measured O157-specific responses of peripheral blood cells through *ex vivo* recall assay. We observed a significantly greater percentage of proliferating lymphocytes from the Adj-Vac compared to NoAdj-Vac (p < 0.05) or NoAdj-NoVac (p < 0.01) groups (Fig. 1A and B). To determine the inflammatory phenotype of T cell subsets, IFNγ positive cells in response to O157 antigen were analyzed (Fig. 1C). Importantly, the CD4 and γδ T cell populations isolated at week 6 from animals in Adj-Vac group were significantly greater in proportion of IFNγ^+^ antigen-specific T cells (p < 0.01) when compared to the NoAdj-Vac group (Fig. 1D).

### Differential production of IL-4 and IL-10 by T cells driven by adjuvant

To fully profile the effector phenotype of T cell subsets induced by different vaccination formulations, the proportion of cells producing IL-10 and IL-4 cytokines in an O157-recall assay were measured. Percentages of γδ T cells isolated from the Adj-Vac group in response to O157-antigen stimulation at week 6 which were positive for IL-4 (4.8 ± 0.9) increased compared to week 0 (0.9 ± 0.5) and those positive for IL-10 (2.5 ± 1.5) decreased compared to week 0 (8.5 ± 3.4) (Fig. 1E, black bars, right panels). Conversely, PBMCs isolated from animals in the NoAdj-Vac group had significantly higher percentages of IL-10-positive CD4^+^ T cells (11.8 ± 1.0) following O157-stimulation compared to week 0 (5.6 ± 0.7) (Fig. 1E, grey bars, left panels). There were no differences in TGF-β or IL-13 secretion in response to O157 antigen stimulation (data not shown).

**Figure 2:**
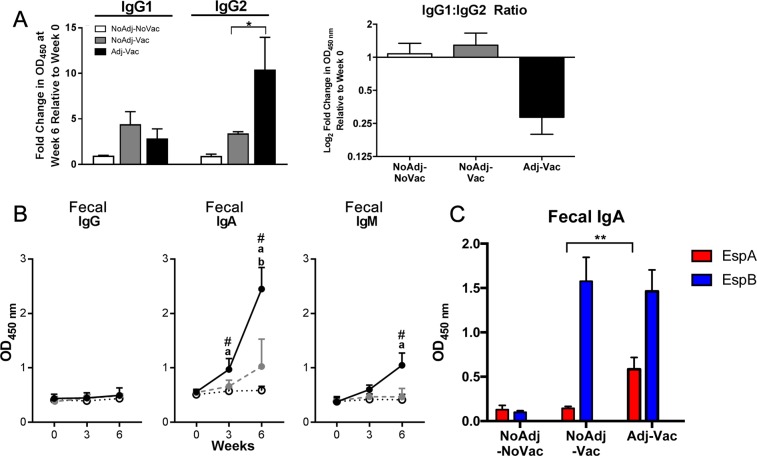
Vaccination increased Th1-biased O157-specific antibodies. (**A**) Measurement of O157-specific IgG1 and IgG2 in the serum (left panel) and ratio of IgG1 and IgG2 (right panel). Samples were measured at week 0 (pre-vaccine) and week 6 (6 weeks post vaccine-prime, 3 weeks post-vaccine boost) and reported as fold change in absorbance at OD_450_ relative to week 0. Antibodies were measured through direct ELISA where plates were coated with an equivalent of 10^8^ cells per well of the formalin-inactivated O157 strain with serum samples diluted 1:1000, and bovine specific IgG1 or IgG2–HRP labeled antibodies diluted 1:50000. Open bars represent non-vaccinated control (NoAdj-NoVac) animals, grey bars represent animals vaccinated with non-adjuvanted vaccine (Adj-Vac), and black bars represent animals vaccinated with the adjuvanted vaccine (Adj-Vac). (**B**) O157–specific fecal IgG (left panel), IgA (middle panel) and IgM (right panel). Samples were measured at week 0 (pre-vaccine), week 3 (3 weeks post-prime vaccine) or week 6 (6 weeks post vaccine-prime, 3 weeks post vaccine-boost). Open circles with dashed line represent non-vaccinated (NoAdj-NoVac) control animals, grey circle with grey dashed line represent animals administered non-adjuvanted vaccine (Adj-Vac), and black circle with solid-black line represent animals vaccinated with the adjuvanted vaccine (Adj-Vac). Antibodies were measured through direct ELISA where plates were coated with an equivalent of 10^8^ cells per well of the formalin-inactivated O157 strain, serum or fecal samples diluted 1:1000, and bovine specific IgG (total), IgA or IgM –HRP labeled antibodies diluted 1:50000, 1:10000, or 1:10000 respectively. Bars are +/−SD with ^a^*p* < *0*.*001* between Adj-Vac and NoAdj-NoVac group; ^b^*p* < *0*.*01* between NoAdj-Vac and NoAdj-NoVac.; ^#^*p* < *0*.*01* between Adj-Vac and NoAdj-Vac group. (**C**) T3SP-specific EspA (red bars) and EspB (blue bars) fecal IgA. Samples were measured at 6 weeks post vaccination-prime (3 weeks post vaccine-boost). Samples were measured by ELISA where plates were coated with either EspA or EspB at 10 μg/mL. Fecal samples were diluted 1:1000 and bovine-specific anti-IgAHRP was diluted 1:10000. Bars are +/−SD with **p<0.001.

### Vaccination with Adj-Vac skewed immune response towards a peripheral O157-specific IgG2 subtype

In order to better characterize immune skewing with the adjuvanted formulation, the subtype of serum O157-specific IgG was analyzed. At 6 weeks post-vaccination, O157-specific IgG2 levels were significantly greater (*p* < 0.01) in serum of Adj-Vac group (10.4 fold increase in OD_450_ from week 0) compared to NoAdj-Vac (3.4 fold increase OD_450_ from week 0) whereas there were no significant differences in IgG1 values (Fig. 2A, left panel). Furthermore, the mean ratio between IgG1 and IgG2 was less than 1 in the Adj-Vac group (0.29 ratio IgG1:IgG2) suggesting that O157-specific IgG response may be skewed towards a Th1-(IgG2) immune phenotype^36,37^ (Fig. 2A, right panel).

### O157-specific fecal IgA was significantly higher in animals in Adj-Vac group

O157-specific IgA detected in the Adj-Vac group increased over the duration of the experiment and levels were at significantly greater (p < 0.001) when compared to the NoAdj-Vac and NoAdj-NoVac group at weeks 3 and 6 post-vaccination (Fig. 2B). Fecal O157-specific IgM levels in the Adj-Vac group were significantly higher when compared to either the NoAdj-Vac or NoAdj-NoVac animals (dashed line, open circles) at week 6 (a, p < 0.001). Overall, there were no significant differences in the amount of fecal O157-specific Ig (regardless of isotype) between the NoAdj-Vac and NoAdj-NoVac groups. Thus, the vaccine required the addition of adjuvant to induce a detectable O157-specific antibody response in the feces.

To evaluate O157 protein specific IgA in feces, ELISAs were performed using recombinant derived EspA or EspB as antigen. A proportion of the fecal IgA-antibody was specific to T3SP EspA and EspB (Fig. 2C). Overall, the data demonstrates that the Adj-Vac group had significantly greater O157-specific IgA (p < 0.001) and greater IgA level to O157 T3SP EspB (p < 0.01) compared to NoAdj-Vac group.

### Fecal extracts prepared from Adj-Vac group, but not NoAdj-Vac group, limited O157 adherence to epithelial cells

**Figure 3:**
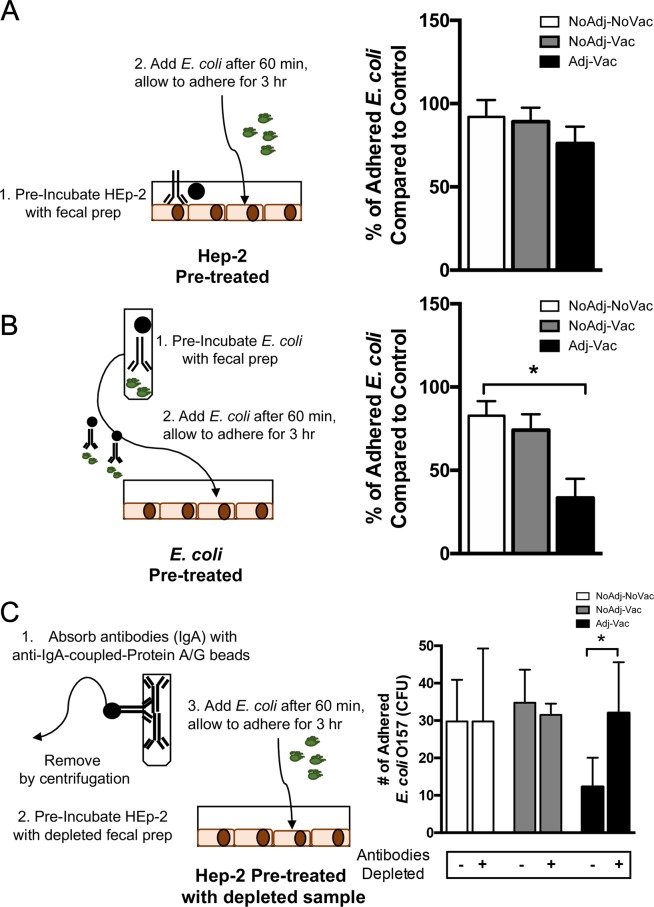
O157 adherence to epithelial cells is greatly reduced with vaccination. HEp-2 epithelial cells (**A**) or O157 cultures (**B**) were incubated with fecal preparations from either mock vaccinated (NoAdj-NoVac), no adjuvant vaccinated (NoAdj-Vac) or adjuvanted vaccinated (Adj-Vac) animals to determine the effects of the fecal extraction samples on *E*. *coli* (O157) adherence to epithelial cells. (**A**) Top panels demonstrate experimental setup where epithelial cells (HEp-2) were incubated with fecal extract preparations (1) and *E*. *coli* added to HEp-2 cells to determine adherence (2). Graphical representation of adherence is demonstrated on right. (**B**) Middle panels demonstrate experimental setup where *E*. *coli* cells (O157) were incubated with fecal extract preparations (1) and *E*. *coli* added to HEp-2 cells to determine adherence (2). (**C**) Bottom panels demonstrate experimental setup where HEp-2 cells were incubated with IgA-depleted fecal extracts (1) and *E*. *coli* added to HEp-2 cells to determine adherence. Graphical representation of adherence is demonstrated on right. Open bars are representive of fecal preparations isolated from mock treated animals (NoAdj-NoVac), grey bars are preparations from non-adjuvanted, vaccinated animals (NoAdj-Vac) and black bars are preparations from adjuvanted, vaccinated (Adj-Vac) animals. Bars are +/−SD. *p < *0*.*01*. Data is representative of 2 experiments.

To determine if fecal extracts contained components that limit O157 adherence to epithelial cells, *in vitro* HEp-2 adherence assays were performed as previously described^23^ and modified slightly to incorporate an adherence blocking step. There were no statistically significant differences between the experimental groups utilizing the HEp-2 cells pre-treated with fecal preparations prior to the addition of O157 (Fig. 3A, right panel). However, a significant decrease (*p* < 0.01) in O157 adherence to HEp-2 cells was observed when O157 was pretreated with fecal extracts isolated from Adj-Vac animals (Fig. 3B, right panel). O157 which was pretreated with fecal extracts remained viable and bacteria were not lysed by other antimicrobial factors (data not shown). Furthermore, by depleting the IgA within the fecal extracts, notably within the vaccinated group (Fig. 3C, black bars), there was a increase in number of adhered O157 compared to samples containing IgA. This data suggests that a soluble factor in the fecal extracts prepared from the Adj-Vac animals, likely O157-specific immunoglobulin, limited O157 adherence and may have contributed to limited colonization and a reduced duration of fecal shedding^38^.

## Discussion

Although Shiga toxin-producing *Escherichia coli* O157:H7 (O157) is pathogenic to humans, within the healthy bovine intestinal tract, O157 colonization occurs without inducing a clinical disease. As ruminants have a large and complex gastrointestinal tract (GIT), which relies primarily on gut microbiota for digestion and fermentation of complex polysaccharides, the bovine GIT requires a dynamic host immune system-bacterial interplay to support a multitude of commensal bacterial species. In humans, for example, non-pathogenic *E*. *coli* strains such as *E*. *coli* strain Nissle 1917 can play an integral role in healthy GIT establishment which can prevent colonization of pathogenic bacterial strains^39,40^ and similar facets of GIT health may apply to cattle. However, development of O157 tolerance may play a role in the immune-modulation of the bovine immune cells of the lamina propria in response to O157^41^. The cattle immune response toward O157 may mimic the human response to Nissle 1917 which is hallmarked by IL-10^42^ and inhibition of inflammatory immune pathways^42,43^. This immune-mediated tolerance and *E*. *coli*-driven immunosuppression of specific immune-mediated inflammatory pathways may be preventing an inflammatory response directed toward O157 strains in cattle.Thus, thwarting these pathways by either eliciting an immune response directed against immune-modulatory or pathogenic traits of O157 could help to reduce colonization of the recto-anal junction (RAJ), which is an important site for O157 colonization^22,44^.

O157 strains produce virulence factors, such as type-3 secretion system proteins (T3SP), which modulate immune responses contributing to O157 immune evasion^45^. T3SP may therefore represent a important targets in driving an immunogenic response. Although many T3SP are injected into cells to modify cellular immune signaling responses, human^46^ and cattle^47^ infection studies have indicated serum specific antibodies directed toward T3SP which include EspA, EspB, Tir and Intimin. Furthermore, T3SP-specific antibodies can be associated with reduced fecal shedding^48^ (reviewed in *Vande Walle et al*.^49^). To this end, development of vaccines that elicit specific antibody and cellular responses against the T3SP immuno-modulatory proteins could potentially reduce colonization and fecal shedding of O157. By reducing O157 fecal shedding in cattle, the potential for human infection could be greatly reduced.

Part of a healthy GIT immune response requires CD4^+^ and γδ T cells to drive anti-bacterial responses targeted toward invading or pathogenic species^50,51^. CD4^+^ T cells in the intestinal lamina propria are an integral part in driving anti-bacterial responses during times of damage to the integrity of the intestinal epithelial barrier^52^. Similarly γδ T cells may either respond to pathogenic bacteria^53^ or play a vital role in the recruitment and homeostasis of lymphocytes and monocytes during an infection setting^54^. γδ T cells are important in supporting the inflammatory cytokine milieu though production of IFNγ and other inflammatory mediators^55^. Some of the bacterial-specific effector T cell populations within the periphery may represent a proportion of the tissue-resident effector or memory populations within the mucosa^33,56^. Therefore, antigen-specific cell populations within the periphery may be an important representation of vaccination-specific cellular responses within the mucosa, serving as a proxy for vaccine elicited immune responses.

As demonstrated here, higher percentages of antigen-specific CD4^+^ and γδ T cells responded to O157 antigen stimulation with IFNγ production after adjuvanted-vaccination. Within the context of O157 colonization and immune activation, murine models of human disease have suggested an important role of IFNγ producing T cells or Th1-skewing immune responses in supporting an inflammatory response directed toward pathogenic O157 at the site of colonization^57^. While O157 may not be pathogenic in cattle, the immune mechanisms limiting colonization may be similar across species. Likewise, γδ T cell-depleted mice demonstrated greater O157 growth within the peritoneal cavity, which the authors suggest is likely due to the loss of γδ T cell-derived cytokines which are important in promoting the survival of CD4^+^ T cells^58^. Furthermore, *in vitro* human and murine studies have suggested that γδ T cell-monocyte interactions, depending on LFA-1/CD11a/CD18 engagement supported by γδ T cell IFNγ cytokine secretion, would direct a robust macrophage activation phenotype able to control pathogenic *E*. *coli* growth^59^. However, the context of peripheral CD4^+^ and γδ T cells as contributing factors in the immunological response to O157 within the bovine host has yet to be fully understood. Transcripts of IFNγ were significantly greater in the RAJ of animals vaccinated and challenged with O157, which was associated with reduced duration and amount of O157 shedding in animal feces^32^. This suggests that T cells producing IFNγ are present at the site of O157 colonization. It is likely that CD4^+^ and γδ T cells contribute to the IFNγ directed response to O157 at the colonization site within the bovine host. Future studies will explore the role of CD4^+^ and γδ T cell subsets specifically within the mucosa of vaccinated animals after O157 challenge to further elucidate the role of these immune cells in protection.

Intestinal IgA responses toward pathogenic bacteria can block bacterial adherence to epithelial cells and play a beneficial role in reduction of bacterial colonization in mouse models of pathogenic O157^60–63^. Similarly, cattle that were immunized against enterotoxigenic *E*. *coli* produced secretory immunoglobulins in milk; which did confer some benefits by protecting against and reducing severity of symptoms of enteropathogenic *E*. *coli* infections on human subjects which consumed the hyperimmunized milk^64,65^. Therefore, in some part, mucosal *E*. *coli*-specific secretory immunoglobulins may play an important role in reducing pathogenic *E*. *coli* colonization, adherence, or clinical pathogenicity^64,65^. One reported vaccination study, which examined EspA of the T3SP complex, demonstrated that EspA-specific IgA did not impact cattle-GIT colonization of O157^66^ but vaccination may reduce shedding through O157-specific IgG, TLR5-ligation or other unknown mechanism^60,67,68^. Similarly, after experimental challenge, levels of fecal IgA specific to O157 LPS or T3SP were highly variable and generally low^69^, although this level of variation may be dependent on a multitude of host factors which impact GIT transit-time^70,71^. However, as demonstrated here, an induction of O157-specific IgA responses within the GIT post-vaccination may represent one mechanism contributing to reduction in O157 adherence to gut mucosa leading to reduced colonization and subsequent shedding from the reservoir host^38^. IgA-containing fecal extracts prepared from vaccinated cattle were able to significantly reduce bacterial attachment to epithelial cells *in vitro*. Although extracts from non-vaccinated controls (NoAdj-NoVac) were able to block approximately 20% of O157 compared to non-treated controls, this is similar to what has been reported with serological studies^72^ and likely represents innate adherence blocking factors or naturally acquired antibodies resulting from previous exposure to *E*. *coli*. Furthermore, when depleting IgA from fecal extracts, the ability of O157 to adhere to Hep-2 cells was recovered, suggesting that an O157-specific IgA responses may represent a previously underappreciated yet critical aspect of blocking O157 adherence to the intestinal tract.

Typically, serum IgG will not be exposed to the intestinal lumen without disruption of the epithelial barrier which would suggest that vaccination-induced serological IgG may not necessarily contribute to continuous barrier protection unlike IgA within the bovine host. Previously, we have reported that serum O157-specific IgG levels were significantly greater (p < 0.001) in the Adj-Vac animals compared to NoAdj-Vac or NoAdj-NoVac animals^19^. However, there was no detectable O157-specific IgG in feces suggesting peripheral IgG is not translocation to the intestinal lumen, at least prior to challenge. Reports suggest an association between serum O157-specific IgG levels and reduced fecal shedding, however a causation relationship between the two has not been clearly delineated^73,74^. With damage at the epithelium, it’s likely that peripheral IgG transudate would reach the epithelium, but it is unclear if this occurs in cattle, or if it occurs after STEC 0157 infection. Here, Adj-Vac induceded a greater IgG2-specific response compared to the other experimental groups (Fig. 2A), suggesting that serum IgG-subtype may be an indicator of the type of immune response elicited by a particular vaccine formulation. Within the context of IgG-subtypes, studies suggest that IFNγ biased (Th1) responses can skew toward antigen-specific IgG2 responses within the bovine host^36,37^. This concurs with the observation examined here, as Adj-Vac generated O157-specific effector T cell subsets which were predominantly IFNγ producing. It is possible that after challenge, disruption of the mucosal-epithelial layer can occur^75^ especially in the neonate^76^, which would allow for IgG into the lumen. It is likely that both antigen-specific IgG and IgA responses are important in limiting O157-shedding from the vaccinated animal, warranting characterization of the O157-specific immunoglobulins in future studies.

Our data demonstrate that vaccination with an adjuvanted, inactivated mutant strain of *E*. *coli*, elicited cellular and humoral (both serum and mucosal) responses directed toward O157. We demonstrated that fecal soluble factors may play a role in controlling bacterial adherence and fecal shedding. In light of these findings, vaccination-directed towards generating inflammatory cellular- and antibody-mediated responses may be able to successfully control transient bacterial colonization within the ruminant reservoir host and can act as predictors of vaccine success. These findings have significant impact on the future development of O157 directed vaccinations within the ruminants and the potential to greatly minimize zoonotic transfer of pathogenic O157 to humans.

## Materials and Methods

### Animals

Samples were derived from a previously reported study^19^. Briefly, twelve clinically healthy, O157-culture negative, Jersey steers of approximately 6 months of age were used for this study. Animals were housed in BSL-2 conditions and fed a maintenance diet of grain and hay. Four animals were randomly assigned to each of the three groups and allowed to acclimate to the housing conditions for 2 weeks prior to start of the experiment. Animals were housed in separate rooms per treatment group. Animals were closely monitored 7 days after vaccination and 3 days after boost for site injection reactions and rectal temperature increase. Animal housing and procedures were approved by the National Animal Disease Center’s Institutional Animal Care and Use Committee (IACUC). All experiments were performed in accordance with IACUC and agency guidelines and regulations.

### Construction of vaccine strain and vaccination schedule

The vaccine strain used in this study was derived by deleting the *stx2* and *hha* genes in O157 strain 6564 and described previously^19^. Briefly, animals (n = 4 per group) were prime vaccinated intramuscularly in the neck region with 2 mL of Adj-Vac, 2 mL of NoAdj-Vac, or mock vaccinated with phosphate-buffered saline (PBS, pH 7.4; (NoAdj-NoVac)). Three-weeks following the first vaccination, animals were given a booster dose of the same vaccine preparation.

### Isolation of Peripheral Blood Mononuclear Cells

10 mL of blood was collected into heparin tubes, red blood cells were lysed utilizing hypotonic salt solution, washed with endotoxin-free PBS (Thermo Fisher Scientific), filtered through a 40 μm cell strainer, counted for viable cells utilizing trypan-blue staining, resuspended in complete RPMI supplemented with 2 mM L-glutamine, 25 mM HEPES buffer, 1% penicillin-streptomycin solution, 50 mg/mL gentamicin sulfate, 1% nonessential amino acids, 2% essential amino acids, 1% sodium pyruvate (all reagents Thermo Fisher Scientific), 50 μM 2-beta-mercaptoethanol (Sigma Aldrich, Saint Louis, MO), and 10% (v/v) Bovine Viral Diarrhea Virus-negative-Fetal Bovine Serum (PAA Laboratories, Etobicoke, ON, Canada) (cRPMI). Peripheral blood mononuclear cells (PBMC) were plated at 10^6^ cells/well into 96-well, tissue culture-treated, round bottom plates.

### Serum Isolation and Preparation of Fecal Extracts

Serum was isolated utilizing coagulation tubes (BD Biosciences, Franklin Lakes, NJ) with centrifugation as previously described^18^. Fecal extracts were prepared as previously described^77^. Briefly, 1 mg fecal samples were washed with 10 mL PBS containing 1% Triton-X. Samples were centrifuged for 30 min at 3200 × *g*. Samples were transferred to 1.8 mL Eppendorf tubes and centrifuged for a second time at 12000 × *g* for 10 min to further clarify samples. Supernatants were filter sterilized using 0.2 μm syringe filters and samples stored at −80 °C.

### Recombinant Protein Generation

Plasmids for protein purification were generated as previously described^20^. Briefly, recombinant plasmids containing a HIS-tag following T3SP sequence (i.e. EspA, EspB) were transfected into *E*. *coli* and single colony selected for protein expression and purification. Liquid medium (Lysogeny broth-Miller containing kanamycin (LB + Kan) (Thermo Fisher Scientific) was inoculated with *E*. *coli* containing the appropriate recombinant plasmid and grown overnight with gentle shaking at 37 °C. Cultures were transferred to 200 mL flasks containing LB + Kan and grown to an OD of 0.6. Cultures were treated with 1 mM IPTG (Sigma Aldrich) to induce HIS-tagged protein generation. Cells were grown for 4 hours at 37 °C, pelleted and froze at −20 °C. Frozen cell pellets were thawed, lysed by detergent buffer containing lysozyme and Benzonase® (Qiagen), centrifuged at 14,000 × *g* for 30 min and the clear lysate was loaded onto nickel-bound columns to isolate his-tagged proteins following manufacturer’s instruction (Qiagen). Purified proteins were quantified with A_280_ and Bradford (BioRad) based colorimetric quantification. Proteins were analyzed for purity using western-blot imaging with anti-His tagged antibody (Thermo Fisher Scientific).

### Enzyme-linked immunosorbent assay (ELISA)

ELISAs were performed as previously described with slight modifications^18^. Briefly, for vaccine-strain specific ELISA, formalin-killed vaccine strain was coated to high binding ELISA plates at an equivalent of 10^8^ cells per well at 4 °C, overnight in bicarbonate buffer (pH of 9.6). For T3SP ELISA, EspA and EspB were coated at 10 µg/mL^20^ at 4 °C, overnight in bicarbonate buffer (pH of 9.6). Coated ELISA plates were washed 5 times with PBS + 0.05% Tween-20 and wells were blocked with StartingBlock (Thermo Fisher Scientific) for 1 h at room temperature. Serum or fecal samples were analyzed in duplicate at three, ten-fold serial dilutions starting at 1:100. Plates were washed 5 times with PBS + 0.05% Tween-20 and Anti-bovine IgA-HRP, IgM-HRP (1:10,000 dilutions) or IgG-HRP, IgG1-HRP, IgG2-HRP (1:50,000 dilution) (Bethyl Labs) were added to plate wells as per manufacturer’s instructions. Ultra-TMB (Thermo Fisher Scientific) was added to plates after 10 washes with PBS + 0.5% Tween-20. Plates were allowed to develop for approximately 5 minutes and stopped with 1M HCl. Plates were immediately analyzed for absorbance at 450 nm (OD_450_) on a SpectraMax Spectrophotometer (Molecular Devices, Sunnyvale CA).

### Adherence Assay and Antibody Depletion

Adherence patterns displayed by O157 strain on HEp-2 cells (human epidermoid carcinoma of the larynx cells with HeLa contamination) (ATCC CCL-23; American Type Culture Collection, Manassas, VA) were determined as described previously^23^. Three protocols were utilized to assay fecal soluble factors on adherence. In assay 1, HEp-2 cells were pretreated with fecal extractions for 60 min. HEp-2 cells were washed with PBS three times prior to the addition of O157. In assay 2, O157 was pretreated with fecal extractions for 60 min. O157 was added to HEp-2 cells and allowed to adhere. In assay 3, Protein A/G microbeads (Sigma Aldrich) were incubated with anti-Bovine IgA (Bethyl Labs) for 1 h at 4 °C and fecal extracts were incubated with coupled-anti-IgA beads for 24 h at 4 °C. Finally depleted fecal extracts were incubated onto HEp-2 cells for 1 h at 37 °C prior to addition of O157. For all assays, cells were incubated with approximately 10^7^ *E*. *coli* O157 cells (OD_600_ = 0.6) for 3 hours and washed 5 times with sterile, room temperature PBS. To release adhered bacteria, HEp-2 cells were gently lysed with 200 μL 0.5% Triton-X solution and duplicate 10 μL, 10-fold serial dilutions of lysates plated on LB + carbinicillin agar plates starting at 10^−4^. After incubation at 37 °C for 24 h, colonies were counted and averaged for number of strongly adherent bacterial cells. Untreated (medium only, mock-treated) HEp-2 cells which were incubated with O157 strain served as baseline colony counts. Data are reported as fold change in number over baseline or in CFUs.

### Flow Cytometry

Doublets, and debris were excluded from all flow cytometry assays on analysis. Dead cells were labeled with a fixable dye following manufacturer’s recommendation (Zombie Live/Dead, BioLegend, San Diego, CA) and dead cells were excluded from analysis. Florescence minus one (FMO) with isotype controls were used for gating and determination of background staining. Heat killed primary cells were labeled with Live/Dead stain and utilized as compensation control for Brilliant Violet 605, Concavalin A (10 μg/mL, Sigma Aldrich) treated cells were stained with proliferation dye only for compensation of Brilliant Violet 421, and manufactured compensation beads (Thermo Fisher Scientific) were used to develop compensation matrix.

### *Ex Vivo* Antigen Stimulation and Recall Response

For recall responses, antigen was prepared from NADC Strain 6564 of Shiga-toxin 2-positive *E*. *coli* O157:H7 (O157). First, O157 was grown in LB medium containing streptomycin. 25 mL of bacterial cells were grown overnight at 37 °C with shaking to an OD^600^ of approximately 1.0. Cells were pelleted and washed 3× in 40 mL PBS using a centrifuge at 3200 × *g*. Cells were heated to 100 °C, frozen at −80 °C for 3 sucessive intervals. Cells were sonicated for 5 minutes at 20 Ω. To verify cells were non-viable, samples were streaked for colonies on LB agar plates where no colonies were observed after 24 h incubation at 37 °C. O157 protein preps were quantified using a Bradford assay following manufacturer’s instruction (Bio-Rad). Protein preparations were diluted to 1 mg/mL concentrations in cRPMI, and aloquotes stored at −80 °C.

PBMCs were plated at 5 × 10^5^ cells/well in 96-well round bottom, tissue culture-treated plates (Falcon). PBMCs were stimulated with either medium-only (mock) or O157 for 72 h.

### Proliferation Assay and Intracellular Staining

Twenty-four hours prior to cell harvest, CellTrace Violet (Thermo Fisher Scientific) was added at 10 μM for visualization of cellular proliferation^78^. For IC staining, at 16 hours prior to harvest, 10 μg/ml brefeldin A (Sigma Aldrich) was added following manufacturer’s recommendations. Cells were harvested and washed with FACS Buffer (PBS containing 1% Bovine Serum Albumin (Sigma Aldrich)) prior to surface and intracellular labeling. Cells were labeled for flow cytometry with primary-targeting antibodies at 21 °C for 15 min in PBS, washed with FACS buffer, and further incubated with appropriate secondary antibodies for an additional 15 min. For intracellular labeling, cells were fixed and permeabilized in saponin/formaldehyde solution (BD Fix/Perm, BD Biosciences) prior to addition of antibodies. After all cellular labeling, cells were resuspended in Stabilizing Fixative (BD Biosciences) and kept at 4 °C until processed. Antibodies used were as follows: mouse anti-bovine γδ TCR (TCR1-N24, δ-chain specific; clone GB21A, isotype IgG2b), surface IgM (clone BIG73A), CD4 (clone IL-A11A, isotype IgG2a), (Washington State University mAb Center, Pullman, WA); CD8-APC labeled (clone CC63), IFN-γ-PE labeled (clone CC302), IL-4-FITC labeled (clone CC303) (Bio-Rad Antibodies, Raleigh, NC); anti-human CD14 (clone M5E2, BioLegend); and anti-bovine IL-10-strepavidin labeled (clone CC320, Novus Biologicals, Littleton, CO). Secondary antibodies used were goat anti-mouse IgG1-AF488, IgG1-allophycocyanin, IgM-AF594, IgM-allophycocyanin, IgG2b-PE-Cy7, IgG2b-AF350, and IgG2b-Cy5 (Southern Biotech, Birmingham, AL) Flow cytometry was performed on an LSR II (BD Biosciences) and analyzed using FlowJo vX (FlowJo, LLC, Ashland, OR) software.

### Statistical Analysis

Data were analyzed with GraphPad Prism7 software (GraphPad Software, La Jolla, CA). Except where noted in the figure legend, statistical analysis performed utilizing one way-ANOVA with multiple comparison of means. *p < 0.05, **p < 0.01, ***p < 0.001.

## Supplementary information

Supplemental Figure 1
