# Supplementary material for: Cellular and Mucosal Immune Responses Following Vaccination with Inactivated Mutant of Escherichia coli O157:H7
Source: Sci Rep. 2019 Apr 22;9:6401. doi: 10.1038/s41598-019-42861-z (PMC6483982; doi:10.1038/s41598-019-42861-z)
Supplement: Supplementary file 1 — Supplemental Figure 1 [file 41598_2019_42861_MOESM1_ESM.pdf]

Cellular and Mucosal Immune Responses Following Vaccination with Inactivated Mutant of *Escherichia coli* O157:H7

Robert G. Schaut, Paola M. Boggiatto, Crystal L. Loving, Vijay K. Sharma

Supplementary Information

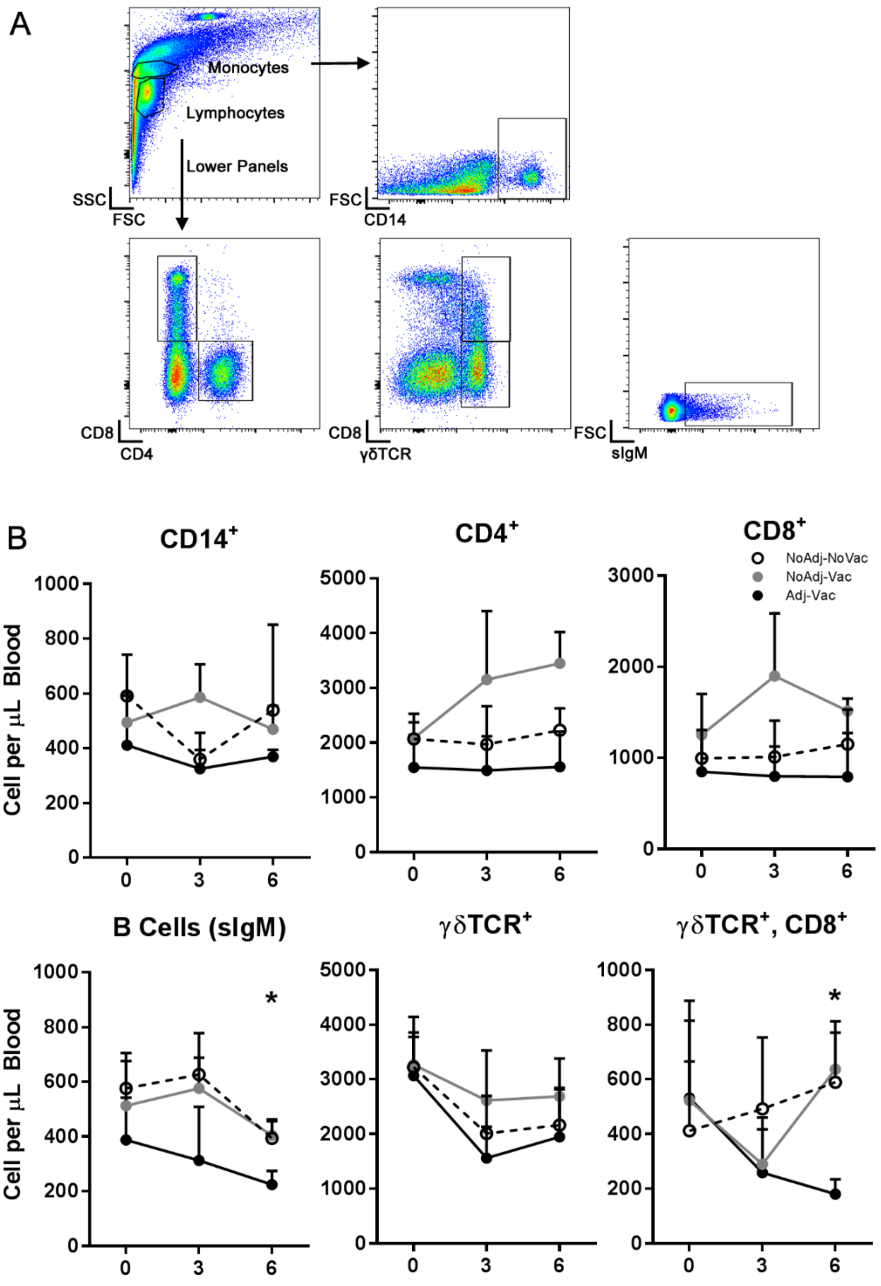

Supplemental Figure 1. Circulating B cells and CD8$^+$ γδ$^+$ cells were reduced in number in animals vaccinated with adjuvanted-vaccine. Whole blood was assayed for levels of CD4, CD8, B cells, CD14, and γδ cells utilizing flow cytometry and standardized counting beads to normalize counts. (**A**) Gating scheme to identify populations. (**B**) Graphical representation of cell populations as indicated. Open circles with dashed line represent mock treated animals (NoAdj-NoVac), grey circles with grey solid line represent non-adjuvanted vaccine animal group (NoAdj-Vac), and black circles with solid-black line represent adjuvanted vaccine animals (Adj-Vac). Samples were

measured at week 0 (pre-vaccine), week 3 (3 weeks post-vaccine, pre-boost) or week 6 (6 weeks post vaccine-prime, 3 weeks post vaccine-boost). Bars represent +SD. * *$p<0.01$* between NoAdj-NoVac and Adj-Vac groups.
